# Enhancer activation from transposable elements in extrachromosomal DNA

**DOI:** 10.1038/s41556-025-01788-6

**Published:** 2025-10-21

**Authors:** Katerina Kraft, Sedona E. Murphy, Matthew G. Jones, Quanming Shi, Aarohi Bhargava-Shah, Christy Luong, King L. Hung, Britney J. He, Rui Li, Seung Kuk Park, Michael T. Montgomery, Natasha E. Weiser, Yanbo Wang, Jens Luebeck, Vineet Bafna, Jef D. Boeke, Paul S. Mischel, Alistair N. Boettiger, Howard Y. Chang

**Affiliations:** 1 Center for Personal Dynamic Regulomes, Stanford University School of Medicine, Stanford, CA USA; 2 Department of Genetics, Stanford University, Stanford, CA USA; 3 Department of Developmental Biology, Stanford University, Stanford, CA USA; 4 Department of Cell Biology, Yale School of Medicine, New Haven, CT USA; 5 Sarafan ChEM-H Institute and Department of Pathology, Stanford University, Stanford, CA USA; 6 Department of Chemical and Systems Biology, Stanford University, Stanford, CA USA; 7 Stanford Cancer Institute, Stanford University, Stanford, CA USA; 8 Basic Science and Engineering Initiative, Stanford Children’s Health, Betty Irene Moore Children’s Heart Center, Stanford, CA USA; 9 Department of Computer Science and Engineering, University of California at San Diego, La Jolla, CA USA; 10 Institute for Systems Genetics, NYU Langone Health, New York, NY USA; 11 Present Address: Amgen Research, South San Francisco, CA USA; 12 Present Address: Max Planck Institute For Molecular Genetics, Berlin, Germany; 13 Present Address: Department of Biology, Massachusetts Institute of Technology, Cambridge, MA USA; 14 Present Address: Department of Neuroscience, Scripps Research, La Jolla, CA USA

**Keywords:** Gene regulation, Cell biology, Cancer

## Abstract

Extrachromosomal DNA (ecDNA) drives oncogene amplification and intratumoural heterogeneity in aggressive cancers. While transposable element reactivation is common in cancer, its role on ecDNA remains unexplored. Here we map the 3D architecture of *MYC*-amplified ecDNA in colorectal cancer cells and identify 68 ecDNA-interacting elements—genomic loci enriched for transposable elements that are frequently integrated onto ecDNA. We focus on an L1M4a1#LINE/L1 fragment co-amplified with *MYC*, which functions only in the ecDNA-amplified context. Using CRISPR-CATCH, CRISPR interference and reporter assays, we confirm its presence on ecDNA, enhancer activity and essentiality for cancer cell fitness. These findings reveal that repetitive elements can be reactivated and co-opted as functional rather than inactive sequences on ecDNA, potentially driving oncogene expression and tumour evolution. Our study uncovers a mechanism by which ecDNA harnesses repetitive elements to shape cancer phenotypes, with implications for diagnosis and therapy.

## Abstract (web summary)

Kraft, Murphy, Jones et al. identify extrachromosomal DNA (ecDNA)-interacting elements (EIEs) enriched for transposable elements within ecDNA in colorectal cancer cells. They show that EIE 14 integrated within ecDNA acts as an enhancer to promote cancer fitness.

## Main

Extrachromosomal DNA (ecDNA) is a prevalent form of oncogene amplification present in approximately 15% of cancers at diagnosis^1–5^. ecDNAs are megabase-scale, circular DNA elements lacking centromeric and telomeric sequences and found as distinct foci apart from chromosomal DNA^6^. Recent work has underscored the importance of ecDNA in tumour initiation and various aspects of tumour progression, such as accelerating intratumoural heterogeneity, genomic dysregulation and therapeutic resistance^7–11^. The biogenesis of ecDNA is complex and tied to mechanisms that induce genomic instability, such as chromothripsis and breakage–fusion–bridge cycles, which are prevalent in tumour cells^6,12–17^.

A key aspect of ecDNA function is their ability to hijack *cis*-regulatory elements that increase oncogene expression beyond the constraints imposed by endogenous chromosomal architecture^18–23^. Consequently, their nuclear organization is tightly tied to their ability to amplify gene expression^18,20^. Likewise, repetitive genomic elements provide a vast network of cryptic promoters or enhancers capable of rewiring gene regulatory networks for proto-oncogene expression—including long-range gene regulation^24–26^. By investigating the three-dimensional (3D) organization of ecDNA, we identified an enrichment of repetitive elements associated with ecDNA structural variation, which we classify as ecDNA-interacting elements (EIEs). We found that insertion of a particular EIE containing a fragment of an ancient L1M4a1 LINE within ecDNA leads to expression of said element that is critical for cancer cell fitness. Our data reveal a relationship between the presence of specific repetitive elements and aberrant expression of oncogenes on ecDNA.

## Results

### ecDNA structural variants enriched for repetitive element insertions

**Fig. 1:**
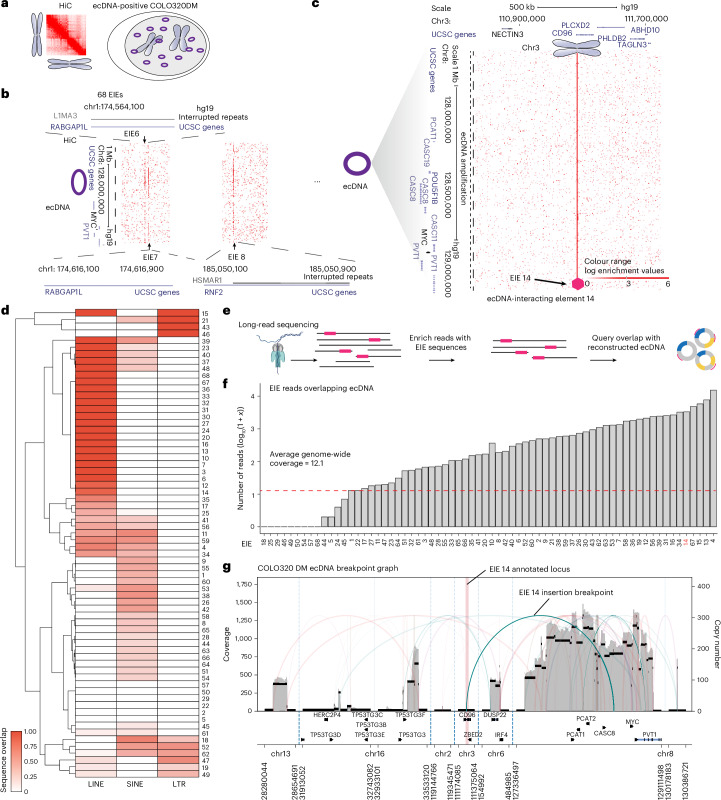
Identification of EIEs. **a**, Schematic of the Hi-C method performed in the ecDNA-containing COLO320DM cell line. **b**, Identification of EIEs. Sixty-eight individual EIEs were manually annotated across all chromosomes based on the interaction across the entirety of the *MYC-*amplified region of chromosome 8. The visualization represents the ecDNA from chromosome 8, with three examples of EIEs localized on other chromosomes. **c**, An example of a specific interaction, EIE 14 on chromosome 3, is enlarged, and associated genes are shown for both loci. The arrow and purple hexagon indicate EIE. **d**, Overlap fraction between EIE sequences and annotated LINE, SINE and LTR elements as reported in RepBase. EIEs are clustered according to similarity in overlap fraction across these three classes of repetitive elements. **e**, Pipeline for using Oxford Nanopore ultralong-read sequencing to identify the overlap of ecDNA genomic intervals and EIE-containing reads. **f**, The number of reads that contain a particular EIE and overlap with an ecDNA interval in the COLO320DM cell line. Counts are reported as log_10_(1 + *x*). Average genome coverage (approximately 12.1) is represented as a dashed red line. **g**, Reconstruction of the ecDNA breakpoint graph for COLO320DM from Oxford Nanopore ultralong-read data using the CoRAL algorithm. The EIE 14 region is highlighted in red, and the breakpoint indicating its translocation to the amplified chromosome 8 locus is annotated. Source data

To interrogate the conformational state of ecDNA, we performed Hi-C on COLO320DM colorectal cancer cells (Fig. 1a). Previous investigation of COLO320DM utilizing DNA fluorescent in situ hybridization (FISH) and whole-genome sequencing identified a highly rearranged (up to 4.3 MB) ecDNA amplification containing several genes, including the oncogene *MYC* and the long non-coding RNA *PVT1*^18,20^. As a large fraction of the ecDNA in COLO320DM is derived from chromosome 8, with smaller contributions from chromosomes 6, 16 and 13, we elected to focus on the chromosome-8-amplified locus containing *MYC* and *PVT1*^20^.

**Extended Data Fig. 1:**
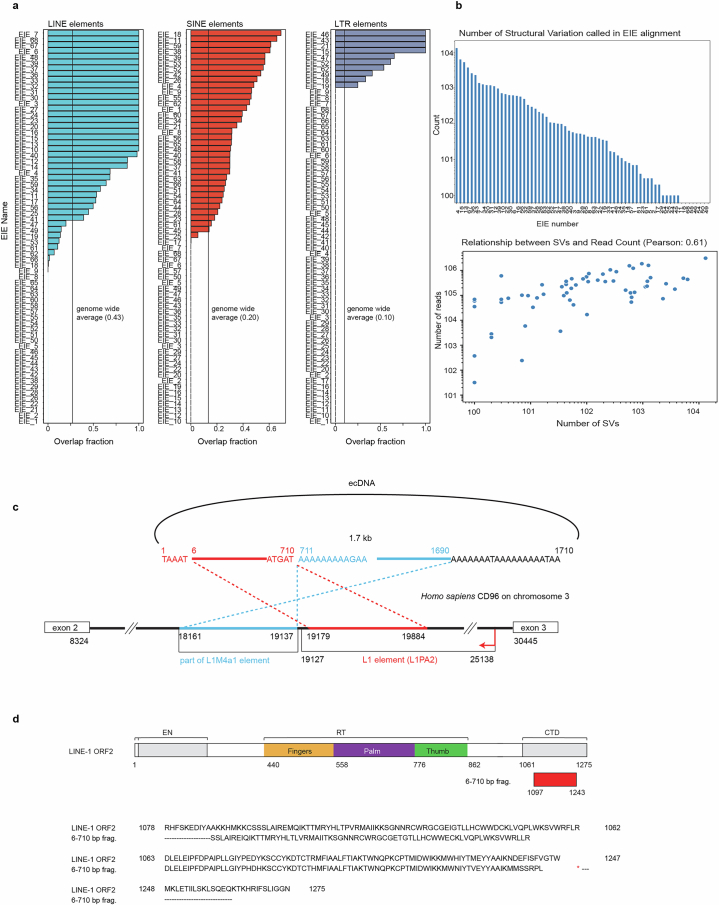
Repetitive-element context, structural variations, and sequence composition of EIE 14. **a**. Overlap of each EIE with the annotated genomic coordinates of LINE, SINE, or LTR elements. The background genome average of each class of repetitive element is reported as a solid black line. **b**. The graph (top) demonstrates the number of structural variations called in stripe alignments. Relationship between structural variations and read count for each element (bottom). Pearson correlation is 0.61. **c**. Schematics of ecDNA harboring 1.7 kb sequence obtained from long-read analysis of EIE 14. The region spanning 6-710 bp shows alignments with 3’ end of the LINE-1 element (L1PA2), whereas the region from 711-1690 bp is notably unique to intron 2 of the *CD96* locus on chromosome 3 (L1M4a1). The L1M4a1-like segment harbors a polyA-signal–like motif (AAAAAG). **d**. Top panel, alignment of predicted protein from 6-710 bp with LINE-1 ORF2 (L1PA2). Bottom panel, amino acids alignment of LINE-1 ORF2 (L1PA2) and 6-710 bp coding protein by clustalW. Source numerical data in source data.

Analysis of the Hi-C maps identified 68 interactions between the chromosome-8-amplified ecDNA locus and other chromosomes that displayed a striking pattern (Fig. 1b and Supplementary Table 1). By binning the data at 1 kb resolution, we found that linear elements in the genome contacted the entirety of the megabase-scale ecDNA amplification in a distinctive stripe (Fig. 1b,c). These contacts were spread across all chromosomes in the genome (Supplementary Table 1). This atypical interaction pattern suggested a complex structural relationship between the chromosome-8-amplified ecDNA and the endogenous chromosome regions (Fig. 1b,c). Further inspection revealed these genomic interactions were enriched for transposable elements (TEs) annotated as LINEs (long interspersed nuclear elements), SINEs (short interspersed nuclear elements) and LTRs (long terminal repeats (Fig. 1d, Extended Data Fig. 1a and Supplementary Tables 2 and 3). As these retrotransposons can acquire the ability to regulate transcription when active, we reasoned that the spatial relationship with oncogenes such as *MYC* may be important for enhanced expression in COLO320DM cells^27,28^. We hereafter refer to these 1-kb interactions, often containing retrotransposons, as EIEs.

While Hi-C is widely used to map genome-wide chromatin interactions, it can also be repurposed to identify structural variants, including rearrangements that are hallmarks of cancer genomes^29,30^. We considered that the atypical striping pattern observed in our Hi-C data was probably the result of structural variation either within the COLO320 genome or due to the insertion of repetitive elements into ecDNA. To discern between these two possibilities, we performed long-read nanopore sequencing (Methods). We chose long-read sequencing to also capture potential heterogeneity in insertion sites in the case of single or multiple integrations (Fig. 1e,f; Methods). We generated median read lengths of 67,000 bp, with the longest read spanning 684,457 bases. Across the 68 EIEs identified, we determined that each participated in a broad spectrum of structural variation—some involved with hundreds or thousands of different rearrangement events (Extended Data Fig. 1b; Methods).

### EIE 14 is a ‘passenger’ on *MYC* ecDNA

After confirming that the identified EIEs were associated with structural rearrangements, we next investigated the overlap between ecDNA and EIE rearrangements. We first reconstructed ecDNA utilizing the CoRAL algorithm^31^, a pipeline that leverages long-read data to accurately infer a set of ecDNAs from the breakpoints (that is, structural variation) associated with amplified regions of the genome (Methods). We found that reads containing EIEs often overlapped ecDNA intervals with greater coverage than expected based on the average genome coverage of our dataset (approximately 12.1), suggesting that these EIEs are present in at least a subset of ecDNA amplifications (Fig. 1e,f). We further investigated CoRAL’s reconstruction of COLO320DM’s complex and heterogeneous *MYC*-containing amplicon and identified a high-confidence breakpoint connecting a chromosome-3-amplified EIE (EIE 14) to an intergenic region between *CASC8* and *MYC* on the chromosome 8 amplification (Fig. 1g; Methods).

We selected this EIE (EIE 14) for further characterization of EIE biology owing to its proximity to *MYC* on the ecDNA and because it contains a segment with homology to L1M4a1, an ancient element distantly related to LINE-1. The percentage of nucleotide conservation of this segment to the L1M4a1 consensus sequence is consistent with L1M4a1’s Kimura divergence value of 34%. We reasoned that this degree of sequence divergence would allow us to specifically target and interrogate its function without unintentionally targeting other repetitive elements in the genome. We also identified a fragment of LINE-1 PA2 and an ORF2-like protein on EIE 14^32,33^ (Extended Data Fig. 1c,d). Although the mechanism generating the adjacency of the fragments remains uncertain, the L1M4a1-like segment harbours a polyA-signal-like motif (AAAAAG), supporting a model in which an L1PA2 transcript reads through its own 3′ end and terminates at this neighbouring signal, producing a 3′-transduced RNA that could be mobilized *in trans* by the LINE-1 enzyme^32,33^ (Extended Data Fig. 1c,d).

**Fig. 2:**
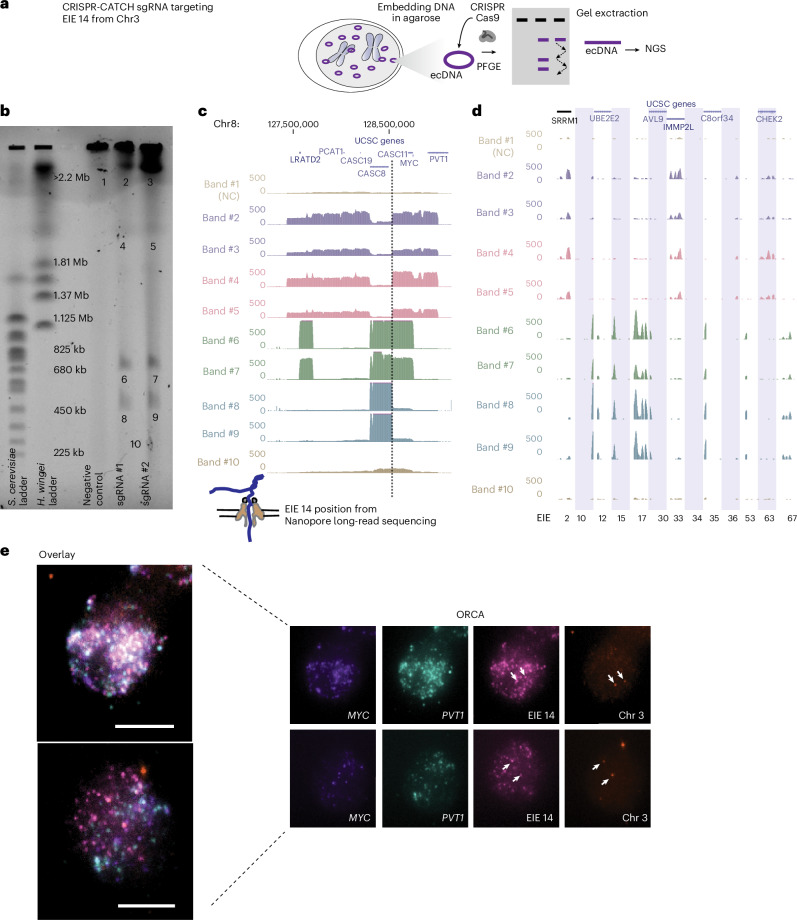
CRISPR-CATCH elucidates ecDNA composition and EIE insertions. **a**, A schematic diagram illustrating the CRISPR-CATCH experiment designed to isolate and characterize ecDNA components. The process involves the use of guide RNA targeting the EIE 14 from chromosome 3. DNA is embedded in agarose, followed by PFGE, allowing band extraction and subsequent next-generation sequencing (NGS) of ecDNA fragments. Negative control (NC) is a guide RNA not targeting ecDNA. **b**, The PFGE gel image displays the separation of DNA fragments, including lanes for the left ladder, ladder, empty lane, negative control, sgRNA #1, sgRNA #2, and band numbers corresponding to those analysed by NGS in in **c** and **d**. Targeting EIE 14 with guide RNAs leads to cleavage of the ecDNA chromosome 8 sequences, resulting in multiple discrete bands and confirming the insertion of EIE 14 onto ecDNA. sgRNA #1 ATATAGGACAGTATCAAGTA; sgRNA #2 TATATTATTAGTCTGCTGAA; full EIE 14 sequences from long-read sequencing are presented in Supplementary Table 6. **c**, Whole-genome sequencing results confirm the presence of EIE 14, originally annotated on chromosome 3, within the ecDNA, between the *CASC8* and *CASC11* genes, approximately 200 kilobases upstream from *MYC*. The dotted line indicates the position of this insertion. Each band is an ecDNA molecule of a different size that contains the EIE 14 insertion. **d**, Additional EIEs identified in the initial Hi-C screen, captured and sequenced in the CRISPR-CATCH gel bands from **b**. Each EIE is represented by a vertical shaded box with genomic coordinates, indicating insertion events within the ecDNA. **e**, ORCA visualization of the COLO320DM cell nucleus. The maximum-projected images show the spatial arrangement of the *MYC* oncogene, EIE 14 and the *PVT1* locus, labelled in different colours for two different cells. The leftmost panel is an overlay of all images registered to nanometre precision (Methods). Scale bars, 5 μm. The Chr3 probe maps to the breakpoints of the EIE 14 origin inside *CD96* intron. Source data

**Extended Data Fig. 2:**
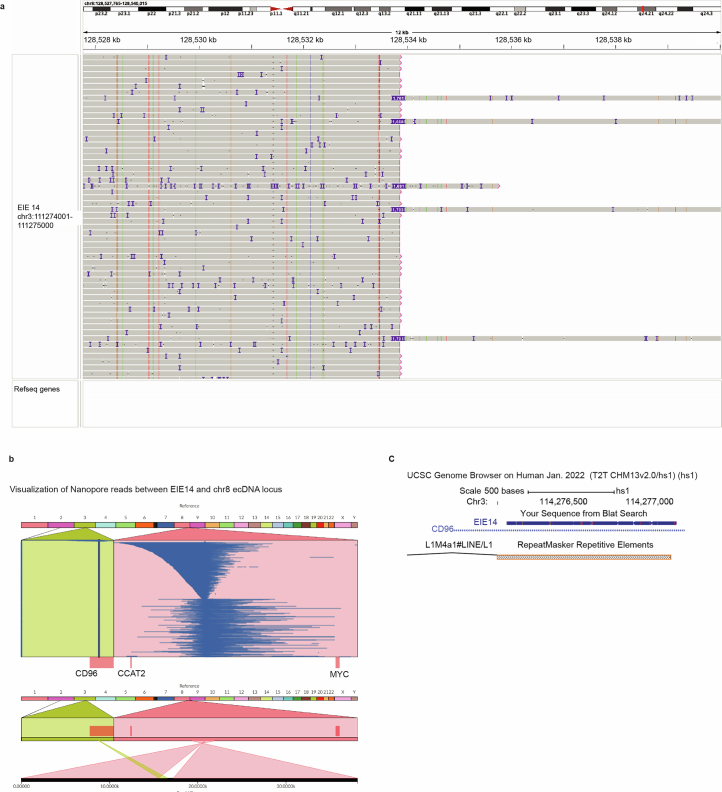
Long‑read DNA sequencing evidence for EIE 14 insertion and genomic context. **a**. Screenshot of the IGV viewer with selected long reads depicting insertion sizes in purple. **b**. Long-read pileup across chr3 and chr8 demonstrating EIE 14 translocation to chr8 ecDNA locus. **c**. Sequence alignment of the T2T genome.

To confirm the computational reconstruction of the ecDNA and the heterogeneity of different ecDNA molecules, we turned to CRISPR-CATCH (Cas9-assisted targeting of chromosome segments)—a method for isolating and sequencing ecDNA—to elucidate the size and variations of ecDNAs containing EIE 14^22^ (Fig. 2a). Targeting EIE 14 with two independent gRNAs, we successfully isolated ecDNA fragments from the COLO320DM cell line for sequencing (Fig. 2b). Sequence analysis of these bands confirmed the presence of EIE 14, originally annotated on chromosome 3, to be inserted onto chromosome 8 between the *CASC8* and *CASC11* genes approximately 200 kilobases away from *MYC*, in agreement with the long-read nanopore sequencing (Figs. 1g and 2c, Extended Data Fig. 2a,b and Supplementary Tables 4–6). Multiple bands of different sizes on the pulsed-field gel electrophoresis (PFGE) gel indicated the presence of varying sizes of ecDNAs, all sharing the EIE 14 insertion within the chromosome 8 amplicon (Fig. 2b,c). Beyond EIE 14, the CRISPR-CATCH approach allowed us to capture and sequence a subset of EIEs initially identified through Hi-C analysis (Fig. 2d). The identification of the additional EIEs observed in the Hi-C data suggest that the ‘striping’ between the ecDNA and endogenous chromosomes is an artefact of these sequences’ presence on ecDNAs, rather than true *trans* contacts, at least for this identified subset. Although the recent T2T genome build^34^ annotates EIE 14 to chromosome 3 (Extended Data Fig. 2c), we found evidence that the structural variant described here between EIE 14 and the *MYC*-containing amplicon region is identified as a translocation event between Chr8:128,533,830 and Chr3:111,274,086 in approximately 46% (minor allele frequency of 0.467646) of individuals without disease^35^ (Supplementary Table 4, row 7). This suggests that this structural variant was preexisting before cancer formation in the COLO320-originating patient and was subsequently amplified as a passenger on ecDNA.

### EIE 14 makes frequent contact with MYC

**Extended Data Fig. 3:**
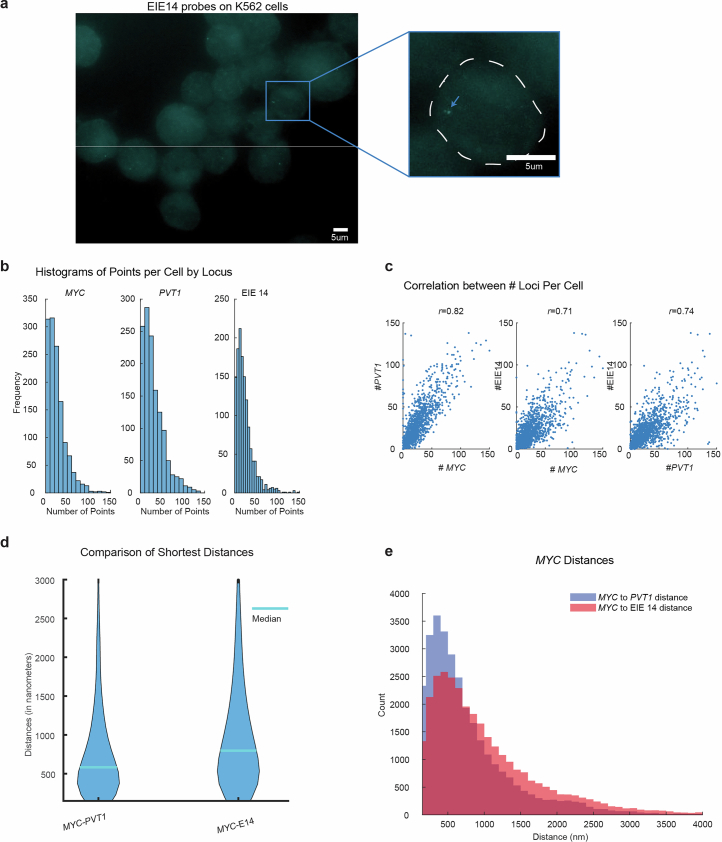
Copy‑number and spatial relationships between MYC, PVT1 and EIE 14. **a**. EIE 14 DNA-FISH labeling of K562 cells lacking ecDNA and amplification of chr8. Arrow points to the FISH signal. The experiment was performed twice with similar results. **b**. Quantification of copy number of *MYC*, *PVT1* and EIE 14 across all measured cells (*n* = 1329, 2 biological replicates). Mean copy number of *MYC* is 29 copies per cell, *PVT1* is 31 copies per cell and EIE 14 is 22 copies per cell. Copies for all species ranged from 0 to 150 copies. **c**. Correlation plots between the loci per cell. Pearson’s correlation coefficient calculated for PVT*1-MYC r* = 0.82, EIE 14*-MYC r* = 0.71, EIE 14*-PVT1 r* = 0.74. **d**. Violin plots of shortest distances of *MYC* to *PVT1* and EIE 14 (median distance denoted by red line). Red line denotes median distance. **e**. Histogram of shortest distances of *MYC* to *PVT1* (blue) and *MYC* to EIE 14 (orange) (Wilcoxon two-sided ranksum *p* = 1.23e^-05). Source numerical data and images are available in source data. Source data

We then utilized Optical Reconstruction of Chromatin Architecture (ORCA) to quantify the spatial relationship of EIE 14 with *MYC*^36,37^ (Fig. 2e). Barcoded probes were designed targeting the unique portion of EIE 14 (1 kb), *MYC* exon 2 (3.1 kb), *PVT1* exon 1 (2.5 kb) and the endogenous chromosome 3 region flanking EIE 14 (3 kb) (Supplementary Table 7) to determine the spatial organization of EIE 14 relative to the ecDNA. These specific exons were chosen to account for the fact that amplicon reconstruction of ecDNA in the COLO320DM cell line demonstrated an occasional rearrangement of *MYC* exon 2 replacement by *PVT1* exon 1 (ref. ^20^). Because EIE 14 is classified as a repetitive element, we confirmed probe specificity by staining the EIE 14 locus in K562 cells that do not contain ecDNA. Indeed, we detect only one to three labelled regions in the non-amplified context (Extended Data Fig. 3a). By contrast, when labelling COLO320DM cells, EIE 14 colocalized with the ecDNA and amplified to a similar copy number per cell (Fig. 2e and Extended Data Fig. 3b). The extensive structural variation detected in the long-read sequencing and the amplification of EIE 14 visualized by ORCA (Extended Data Fig. 3b) suggest a model in which the element resides in the sequence amplified on ecDNA and participates in *cis* and/or *trans* contacts with other ecDNA molecules.

**Fig. 3:**
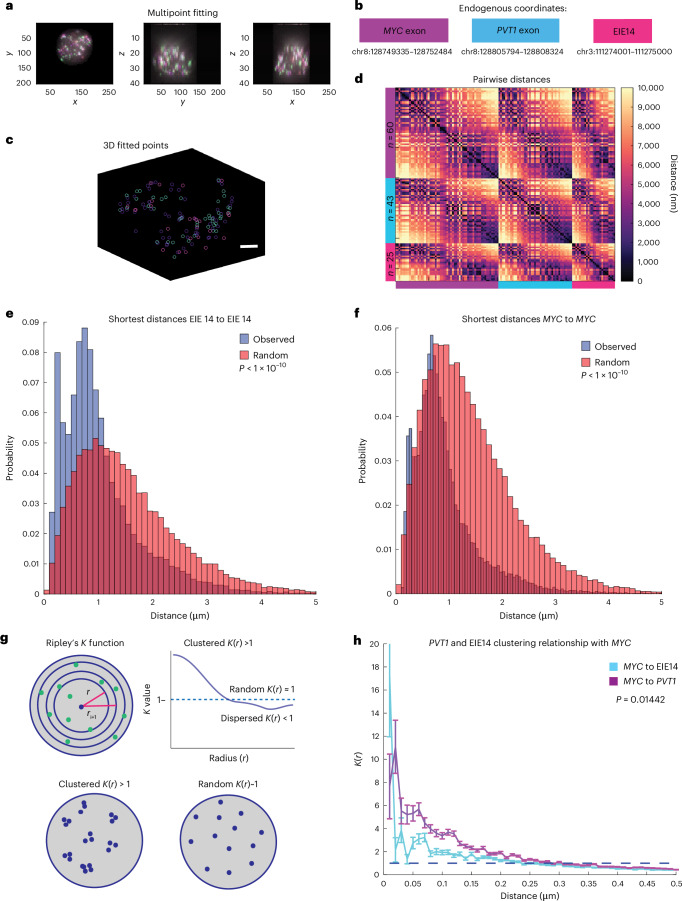
EIE 14 spatially clusters with *MYC.* **a**, *x*,*y*,*z* projections of *MYC* exon (purple), *PVT1* (blue) and EIE 14 (pink). **b**, Endogenous coordinates of all three measured genomic regions. **c**, Single-cell projection of the 3D fitted points from **a**. Scale bar, 2 μm. **d**, Pairwise distances between *MYC* (purple), *PVT1* (blue) and EIE 14 (pink) of a single cell. Number of fitted points per genomic region *n* = 60*, n* = 43 and *n* = 25, respectively. **e**, Histogram showing the distribution of observed shortest pairwise distances between EIE 14 signals, compared with the expected shortest pairwise distances from randomly simulated points within a sphere (two-tailed Wilcoxon ranksum *P* < 1 × 10^−10^) of *n* = 1,329 analysed cells across two biological replicates. **f**, As in **e** but for *MYC*-to-*MYC* shortest pairwise distances (two-tailed Wilcoxon ranksum *P* < 1× 10^−10^). **g**, Schematic of Ripley’s *K* function to describe clustering behaviours over different nucleus volumes. Top: the nucleus divided into different shell intervals and how the *K* value is plotted for increasing radius (*r*). Bottom: an example of what clustered *K*(*r*) *>* 1 versus random *K*(*r*) *≈* 1 points could look like. *K* values greater than one indicate clustering behaviour relative to a random distribution over that given distance interval (*r*), *K* values of ~1 denote random distribution, while *K* values less than 1 indicate dispersion behaviour. **h**, The average *K*(*r*) value across distance intervals of 0.01–0.5 μm in 0.02-μm step sizes to describe the clustering relationship of *PVT1* and EIE 14 relative to *MYC* across different distance intervals (μm). Error bars denote s.e.m. (two-tailed Wilcoxon ranksum *P* = 0.01442). Source data

It has been proposed that amplified loci within ecDNA are able to regulate oncogene expression through *cis* interactions on the same ecDNA molecule as well as *trans* interactions between ecDNAs via a clustering mechanism^20^. As such, it is important to understand not only the structural variations of ecDNA, but also their spatial organization within the nucleus to gain a comprehensive understanding of their potential regulatory functions. We quantified the spatial distributions of *MYC* exon 2, *PVT1* exon 1 and EIE 14; the imaged loci were fitted in three dimensions with a Gaussian fitting algorithm to extract *x*,*y*,*z* coordinates (Fig. 3a–c; Methods). The copy number of identified loci varied from zero detected points to 150 per cell. On average, *MYC* had 29, *PVT1* had 31 and EIE 14 had 22 copies per cell (Extended Data Fig. 3b). Similar distributions of points per cell, as well as strong correlation (*r* > 0.7) between the number of points per loci per cell (Extended Data Fig. 3c), suggest that this EIE is not inserted into multiple sites on a single ecDNA.

Once the centroids of each point per cell were identified (Fig. 3c), we calculated the all-to-all pairwise distance relationship (Fig. 3d). The off-diagonal pattern of distances between EIE 14, *MYC* and *PVT1* suggested a tendency for these loci to cluster at genomic distances <1,000 nm. We further quantified the spatial relationships across all 1,329 imaged cells by calculating the shortest pairwise distances between the three loci. To determine if these ecDNA molecules were spatially clustering in cells, we leveraged our observation that each ecDNA molecule carries a single copy of *MYC* and *EIE 14*. Thus, distances between *MYC* and other *MYC* loci should be closer than random if the ecDNA were spatially clustered. Random distances were simulated in a sphere with the identical number of points per a given cell. The distribution of shortest pairwise distances between *MYC* and *MYC* and between *EIE 14* and *EIE 14* were left-shifted compared with the randomly simulated points, suggesting a non-random organization (Fig. 3e,f, *P* < 1 × 10^−10^). The median observed versus expected distances between each *EIE 14* loci were 748 nm and 927 nm, respectively, and the median observed versus expected distances between each *MYC* loci were 707 nm and 814 nm, respectively.

Previous work has proposed that enhancers can exert transcriptional regulation on promoters at a distance of up to 300 nm via accumulation of activating factors^38–41^. To determine whether EIE 14 and *MYC* are within this regulatory distance range on ecDNA molecules, we calculated the pairwise distances between loci. Although the median distances between *MYC* and EIE 14 (797 nm) and *PVT1* (585 nm) were greater than 300 nm, 12% and 20% of these loci, respectively, were within the regulatory range of *MYC* (Extended Data Fig. 3d,e).

To investigate the spatial relationship between EIE 14 and *MYC* while controlling for locus density, we calculated the degree of spatial clustering across distance intervals using Ripley’s *K* spatial point pattern analysis (Methods; Fig. 3g)*. MYC* exhibited the strongest clustering with *EIE 14* at distances less than 200 nm (*K* value >1), and this behaviour approached a random distribution at greater distances (*K* value ~1; Fig. 3g,h). On average, the distances between *MYC* and EIE 14 were greater than those between *MYC* and *PVT1*. However, at distances below 300 nm, EIE 14 and *PVT1* displayed similar clustering behaviour with *MYC* (Fig. 3h and Extended Data Fig. 3d,e). This clustering suggests that EIE 14 is acting as a proximity-dependent regulator of *MYC* reminiscent of enhancer–promoter interactions^42^. Altogether, the spatial clustering behaviour of this ecDNA species measured here and previously^20^, the propensity for *MYC* to engage in ‘enhancer hijacking’^43^ and the ability of reactivated repetitive elements to engage in long-range gene activation^27^ suggest that any genomically linear separation of *MYC* and EIE 14 is overcome in both *cis* (interaction with *MYC* on the same ecDNA molecule) and *trans* (ecDNA–ecDNA interactions).

### EIE 14 is critical for cancer cell fitness and displays enhancer activity

**Fig. 4:**
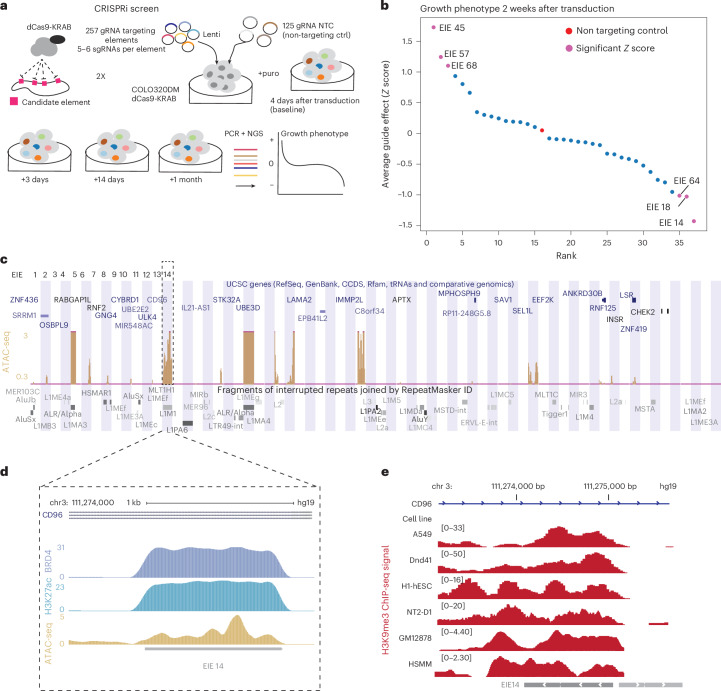
EIE 14 is important for cell proliferation and has enhancer signatures. **a**, Schematic of the CRISPRi screening strategy used to evaluate the regulatory potential of the 68 EIEs by designing 4–6 gRNAs per element for a total of 257 genomic regions tested and 125 non-targeting control sgRNAs. The screen involved the transduction of cells with a lentivirus expressing dCas9-KRAB and the sgRNAs such that each cell received one sgRNA, followed by calculation of cell growth phenotype over a series of timepoints (baseline (4 days), baseline + 3 days, baseline + 14 days and baseline + 1 month). The screen was further filtered on guide specificity (Methods), and 36/68 targeted EIEs met the qualifying threshold. **b**, The growth phenotype of COLO320DM cells 2 weeks post-transduction, relative to NTC. Each point represents the average guide effect (*Z* score) for sgRNAs targeting the 36 qualifying EIEs, ranked by their impact on cell growth. EIE 14 is indicated by dashed rectangle with negative *Z* score <−1 (significant negative impact on cell viability). See Extended Data Fig. 4 for additional timepoints. Positive hits are labelled in pink with their corresponding EIE. **c**, UCSC Genome Browser multiregion view showing the locations of the EIEs within the genome. Each EIE is indicated by a vertical bar. The browser displays the annotations for genes and repetitive elements such as *Alu*, LINE and LTR elements (RepeatMasker); the ATAC-seq dataset^20^ is normalized for library size (Methods). **d**, Zoom-in of EIE 14’s histone marks: enrichment of H3K27 acetylation^18^, BRD4 binding^20^ and ATAC-seq peaks. ChIP-seq data were normalized to input to control for copy number. ATAC-seq data were normalized to library size (Methods). **e**, H3K9me3 histone modification of EIE 14 across ENCODE cell lines^50,51^.

**Extended Data Fig. 4:**
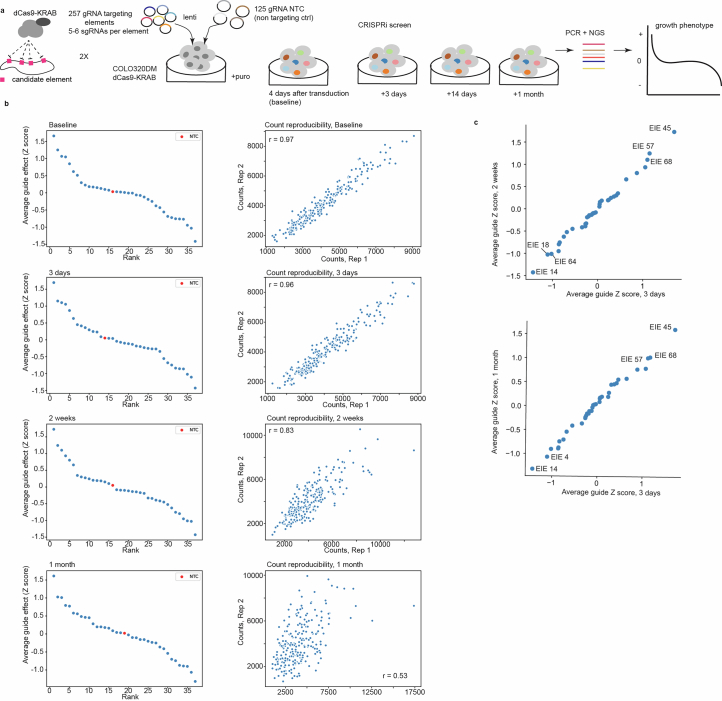
CRISPRi screen of EIEs in COLO320DM for different timepoints. **a**. Schematic of the CRISPRi screening strategy used to evaluate the regulatory potential of the 68 EIEs by designing 4-6 gRNAs per element for a total of 257 genomic regions tested and 125 non-targeting control sgRNAs. The screen involved the transduction of cells with a lentivirus expressing dCas9-KRAB and the sgRNAs such that each cell received 1 sgRNA, followed by calculation of cell growth phenotype over a series of time points (Baseline(4 days), Baseline + 3 days, Baseline + 14 days, and Baseline + 1 month). The screen was further filtered on guide specificity (methods) and 36/68 targeted EIEs met the qualifying threshold. **b**. The growth phenotype of COLO320DM cells and reproducibility of counts between two biological replicates at different timepoints for the 36 qualifying EIEs. Each point represents the average guide effect (*Z*-score). Pearson correlations (*r*) are reported for reproducibility plots. **c**. Growth phenotypes of the qualifying EIE-targeting guides in COLO320DM cells across multiple time points. Each point represents the average guide effect (*Z*-score) for sgRNAs targeting a specific EIE. Guides with an average abs(*Z-*score) > 1 are annotated.

To test whether the identified TEs are important for the cancer cell proliferation, we performed a CRISPR interference (CRISPRi) growth screen targeting a subset of EIEs in COLO320DM cells engineered to stably express dCas9-KRAB^44^ (Fig. 4a,b). We were able to target 36 out of the 68 EIEs with single guide RNAs (sgRNAs) that met the following criteria: (1) must meet stringent specificity criteria to reduce potential off targets intrinsic to repetitive sequences (Methods) and (2) have at least two sgRNAs per EIE. We also included 125 non-targeting controls (NTC) that were introduced into cells with the EIE sgRNAs via lentiviral transduction (Supplementary Table 10). After transduction, we monitored cell proliferation at multiple timepoints: 4 days (baseline), 3 days after baseline, 14 days and 1 month (30 days), followed by deep sequencing to quantify sgRNA frequencies (Fig. 4b). We obtained highly reproducible guide counts across replicates and timepoints (Extended Data Fig. 4b,c).

Our data showed that the growth phenotype curve for 3 out of 36 of our targeted EIEs at various timepoints indicated a *Z* score of less than −1, which suggested a significant negative impact on cell viability, with an acute growth defect after only 3 days (Fig. 4b, Extended Data Fig. 4 and Supplementary Tables 8 and 9). These elements were categorized as evolutionarily older based on their retrotransposition activity in the human genome and spanned classes (LINEs, SINEs and LTRs) (Supplementary Table 11). The enrichment of old TEs may be confounded by the relative ease of targeting sequences with increased sequence divergence. They are generally found in gene-poor regions, making it unlikely that silencing would lead to secondary effects from heterochromatin spreading. Collectively, these results suggest that a subset of our targeted EIEs, including EIE 14, can contribute to cancer cell growth and fitness. We speculate that this is related to EIE interaction with *MYC*, as knockdown of this oncogene has been shown to have similar effects on COLO320DM growth and survival^45,46^. In addition, 3 out of 36 of the measured EIEs also had a *Z* score greater than 1, indicating a significant increase of cell growth or fitness. The identity of these elements also spanned multiple element classes, with two (EIE 68 and EIE 45) located within uncharacterized non-coding RNAs, and one (EIE 57) within the first exon of the ANKRD30B protein-coding gene, which has been implicated in cell proliferation^47^. Further investigation of these hits is warranted in future studies to explain their positive effects on cell growth, especially those within the uncharacterized non-coding RNA regions.

**Extended Data Fig. 5:**
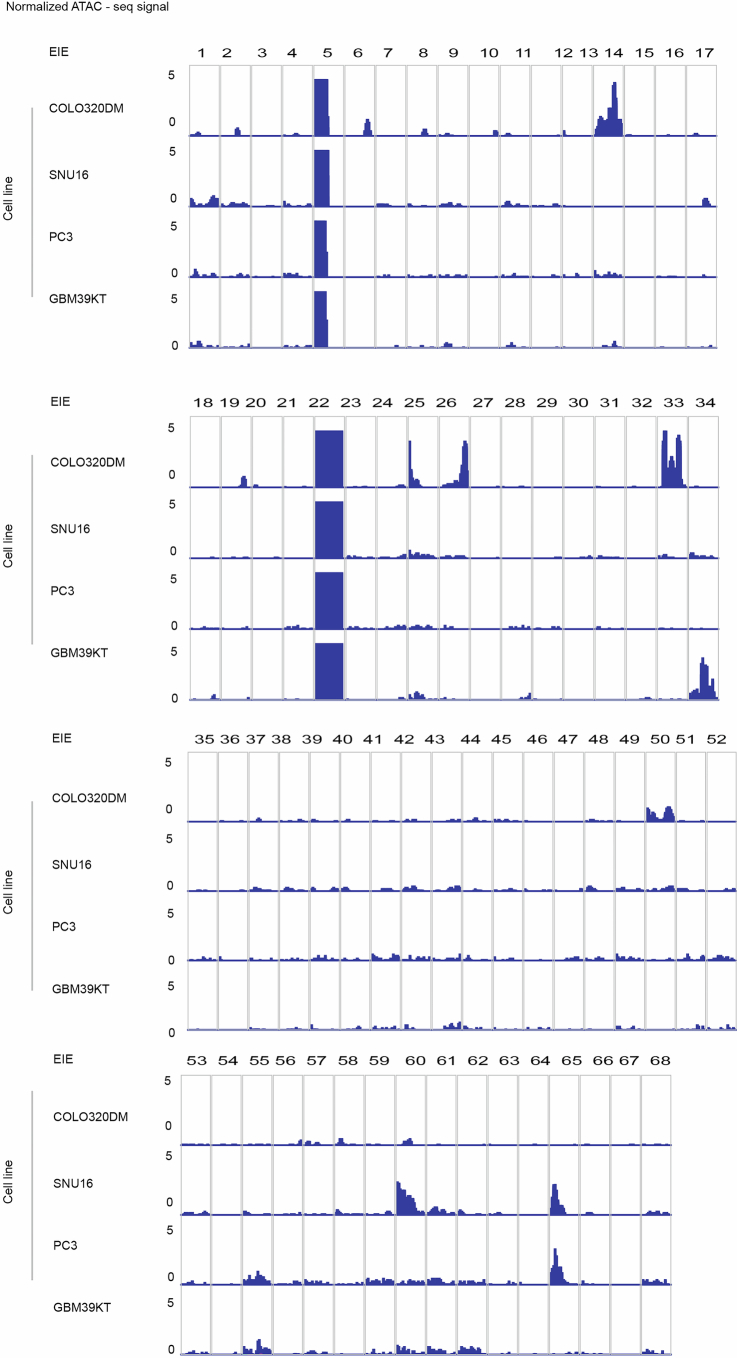
ATAC‑seq signal of EIEs across cell models. ATAC-seq data for COLO320DM and SNU16 was obtained from Hung, Yost et al.^20^ and for PC3 and GBM39KT from Wu et al.^18^. Previously, ATAC-seq data was mapped to hg19. ATAC-seq data was further normalized by library size.

The strongest growth defect was observed for perturbation of EIE 14 (Fig. 4b), which when combined with our finding of its co-localization with ecDNA-amplified *MYC* (Fig. 3h), suggests a potential enhancer-like regulatory role for this EIE. To examine the epigenetic landscape of this element we leveraged copy-number-normalized chromatin immunoprecipitation sequencing (ChIP-seq) measuring H3 lysine 27 acetylation (H3K27ac), BRD4 occupancy and assay for transposase-accessible chromatin using sequencing (ATAC-seq) accessibility data. These epigenetic features are all commonly associated with enhancer activity^18,48,49^. Notably, many EIEs, including EIE 14, were accessible in COLO320DM cells (Fig. 4c,d and Extended Data Fig. 5). The measured accessibility of EIE 14 contrasts the normally silenced H3 lysine 9 trimethylation (H3k9me3) state across annotated human cell lines^50,51^ (Fig. 4e). Cross-referencing our identified EIEs with accessibility data from other ecDNA-containing cell lines demonstrated that accessibility of EIEs is a more generalizable phenomenon beyond COLO320DM cells (Extended Data Fig. 5). Altogether, the accessibility and proximal clustering of EIE 14 points towards active regulatory potential of this element in COLO320DM cells, while identification of accessible EIEs across cell lines suggests a broader functional relevance of EIE regulatory potential on ecDNA^48,49^ (Extended Data Fig. 5).

**Fig. 5:**
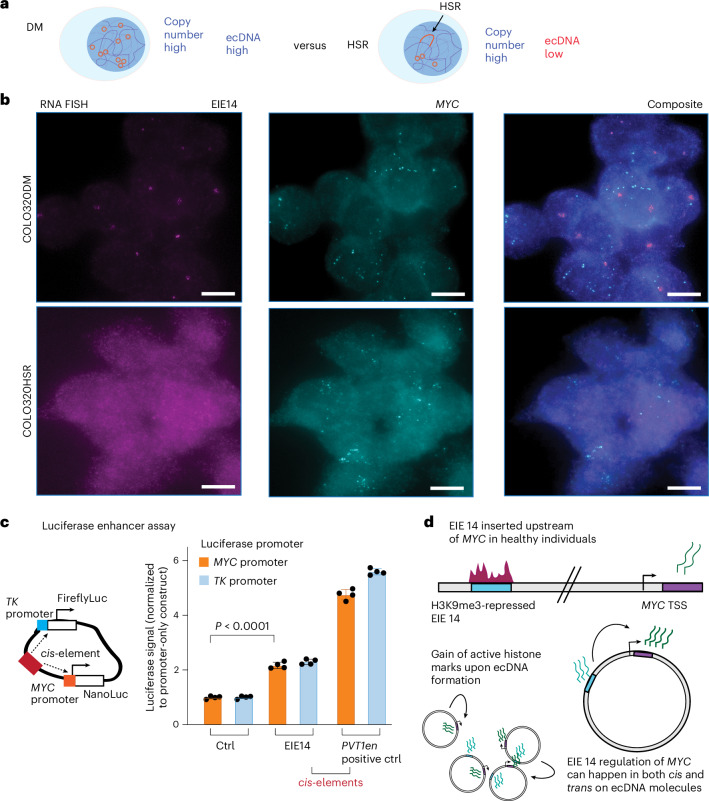
ecDNA context is critical for EIE 14 enhancer activity. **a**, Schematic outlining the COLO320DM cell line as having high copy number and high ecDNA levels, versus the HSR cell line, which has high copy number but low ecDNA. **b**, RNA-FISH labelling for EIE 14 and *MYC* exon 2 transcription in COLO320 DM and HSR. Median transcripts for EIE 14 are 4 and 0 for the DM and HSR cells (two-tailed Wilcoxon ranksum *P* = 8.22 × 10^−94^), respectively. DM cells have a median of 14 *MYC* transcripts, and HSR cells have a median of 8 transcripts per cell (two-tailed Wilcoxon ranksum *P* = 2.18 × 10^−66^). *n* = 712 cells (DM) *n* = 681 (HSR) across two biological replicates. Scale bars, 8 μm. **c**, Luciferase enhancer assay schematics and fold change in luciferase signal driven by either *MYC* or TK promoter normalized to promoter-only construct. *n* = 4 biological replicates. EIE 14 compared with positive control (*PVT1* positive control from ref. ^20^). *P* values obtained from two-tailed unpaired *t*-test. Error bars are standard deviations from the mean. **d**, Schematic outlining EIE 14 as a translocation event in healthy patients where EIE 14 is normally inactive across annotated cell lines (see **a**). EIE 14 gains regulatory potential when it is amplified within ecDNA as a consequence of translocation near *MYC*. EIE 14 can then act as a regulator of *MYC* in both *cis* and *trans* contacts within and between ecDNAs. Source data

**Extended Data Fig. 6:**
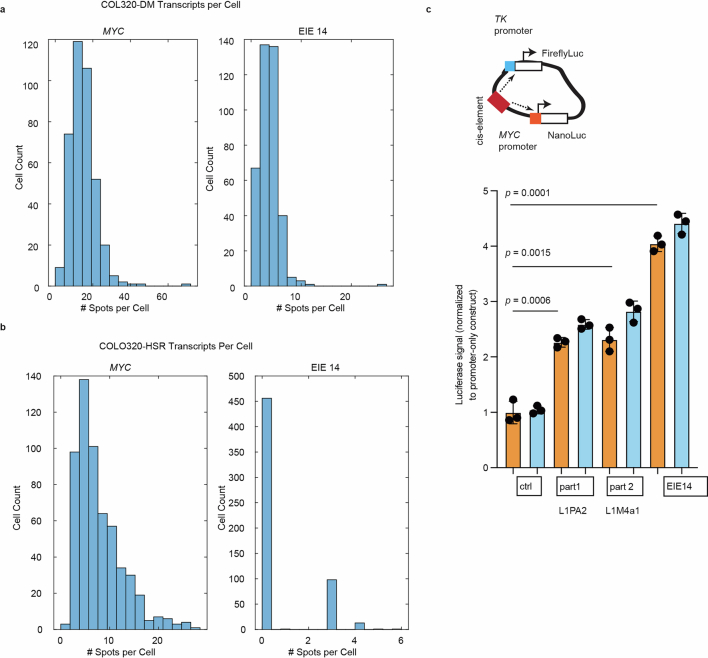
RNA‑FISH quantification and enhancer reporter activity for EIE 14. **a**. Quantification of RNA-FISH signal on a per cell basis from COLO320-DM cells labeling *MYC* exon 2 and EIE 14 (see Methods for quantification method). *n* = 712 cells across 2 biological replicates. **b**. As in (**a**) but for COLO320-HSR cells. *n* = 681 cells across 2 biological replicates. **c**. Fold change in luciferase signal driven by the L1PA2 (part 1), L1M4a1 (part2) and combined (EIE14) regions of EIE 14 for *n* = 3 biological replicates. *P*-value *MYC* promoter (ctrl) vs part 1 *p* = 0.0006; *p*-value *MYC* promoter (ctrl) vs part 2 *p* = 0.0015; value *MYC* promoter (ctrl) vs part 1+part2 (EIE14) *p* = 0.0001 (*p-*values obtained from two-tailed unpaired t-test). Error bars are stanford deviations from the mean. Source numerical data and images are available in source data.

To determine whether EIE 14 activity is a consequence of ecDNA formation, we performed RNA-FISH on the sequence-specific 1-kb segment of EIE 14 in COLO320DM and isogenic COLO320HSR cells. The homogeneously staining region (HSR) cell line contains a similar copy number amplification of the *MYC*-amplified portion of chromosome 8, but the majority of these copies have integrated into chromosomes^18^ (Fig. 5a). We reasoned that, if the unique extrachromosomal context of ecDNA facilitates activation of EIE 14, we should not see evidence of its activity in the COLO320HSR genome-integrated context. Indeed, we observed distinct transcription events in the COLO320DM line (median *n* = 8 transcripts per cell) but not in the HSR line(median *n* = 0 transcripts per cell; Fig. 5b and Extended Data Fig. 6a,b).

Finally, to directly test the ability for the EIE 14 sequence to act as an enhancer of *MYC* expression, we performed a luciferase reporter assay measuring its ability to activate transcription of *TK* and *MYC* promoters^20,52^ (Fig. 5c). EIE 14 significantly increased *MYC* promoter-mediated reporter gene expression relative to the promoter-only control, signifying bona fide enhancer activity (Fig. 5c). Separating EIE 14 into L1M4a1 and L1PA2 fragments further demonstrated that both sequences can individually act as enhancers, with an additive effect when combined (Extended Data Fig. 6c). In sum, the enhancer-associated features and regulatory activity of the luciferase assay suggested that EIE 14, and possibly other EIEs, have been co-opted as regulatory sequences when found on ecDNA, influencing the expression of ecDNA-borne oncogenes (Fig. 5d).

## Discussion

This study uncovers a mechanism by which TEs, typically silenced by heterochromatin, may acquire regulatory potential when amplified on ecDNA^53–55^. Somatically active retrotransposition events^56^, as induced by LINEs and SINEs, are abundant in the human genome and represent a major source of genetic variation^57^. Across cancer types, retrotransposon insertions contribute significantly to structural variation, genomic rearrangements, copy number alterations and mutations—including in colorectal cancer^58–65^. The activity of these elements in cancer can induce genomic instability and drive the acquisition of malignant traits. For instance, when reactivated LINE-1 elements are inserted into the APC tumour suppressor gene in colorectal cancer, they disrupt gene function and confer a selective advantage^66^. In other contexts, TEs act as bona fide transcriptional enhancers, amplifying oncogenic gene expression and promoting tumorigenesis^26^.

Here, we describe the enhancer-like activity of a specific identified element, EIE 14, which becomes active through its association with ecDNA (Fig. 5d). ecDNAs, which are randomly segregated during cell division, are subject to strong selective pressure^10^. The recurrent co-amplification of TEs on ecDNA-containing cell lines suggests they may contribute to ecDNA fitness and oncogenic function. We show that retrotransposons such as L1M4a1/EIE 14 can escape the inactive chromatin environment of their native genomic loci when inserted within the transcriptionally permissive landscape of ecDNA^18^. In fact, we demonstrate that EIE 14 is transcriptionally active only in the context of ecDNA and not in the endogenous chromosomal context of the copy-number-matched, isogenic COLO320HSR cells. The context-specific transcription suggests a purely epigenetic regulation imbued by the local environment of ecDNA. This environment enables EIE 14 to potentially influence nearby oncogenes such as *MYC*. Given that LINEs have been shown to exhibit enhancer-like behaviour when reactivated^27,28,67^, the clustering of ecDNA molecules observed through ORCA may further enhance spatial feedback^68^ of both *cis*- and *trans*-regulatory interactions of EIE 14 with oncogenic targets.

Although EIE 14 is incapable of autonomous transposition and lacks a complete L1M4a1 sequence, its activity following integration into ecDNA suggests that degenerate ancient sequences can become functionally active under appropriate conditions. Previous work has shown that single-nucleotide polymorphisms associated with familial cancer risk often affect the biochemical activity of noncoding enhancer elements linked to oncogenes activated in cancer^69,70^. Our results extend this model by proposing that inherited variation in ancient TE insertions, such as EIE 14 near *MYC*, can create latent enhancers that become activated when the oncogene locus is excised into ecDNA.

Perturbation of EIE 14 through CRISPRi resulted in impaired cell growth in COLO320DM cells, indicating that its reactivation contributes to the colorectal cancer phenotype. Quantifying the precise downregulation of *MYC* is constrained by ecDNA heterogeneity, a narrow temporal window in MYC-addicted cells, rapid growth arrest and subsequent loss of successfully targeted cells. While this functional evidence supports a potential oncogenic role, further studies focusing on in vivo analyses are necessary to determine whether TEs on ecDNA are sufficient to confer a survival advantage or correlate with poor patient prognosis. Notably, recurrent LINE-1 amplification on ecDNA has been observed in primary oesophageal cancer, providing in vivo support for the clinical relevance of this phenomenon^71^.

Finally, the amplification of retrotransposable elements onto ecDNA introduces a mechanism that increases ecDNA structural variation by leveraging the approximately 40% of the genome composed of typically silenced repetitive elements. Retrotranspositions are, in fact, the second-most frequent type of structural variant in colorectal adenocarcinomas^72^. Just as transposons have played a major role in bacterial plasmid evolution through cycles of insertion and recombination^73^, our findings suggest a parallel evolutionary trajectory in human oncogenic ecDNAs. The transcriptionally permissive state of ecDNA enables these elements to potentiate oncogene activation and selection—making them both prognostic biomarkers and potential therapeutic targets.

## Methods

### Cell culture

Cell lines were obtained from ATCC. COLO320DM (CCL-220) and COLO320-HSR (CCL-220.1) cells were maintained in RPMI; Life Technologies, cat. no. 11875-119 supplemented with 10% foetal bovine serum (Hyclone, cat. no. SH30396.03) and 1% penicillin–streptomycin (Thermo Fisher, cat. no. 15140-122). All cell lines were routinely tested for mycoplasma contamination. The presence of ecDNA in cell lines was confirmed via metaphase spreads.

### Hi-C

Ten million cells were fixed in 1% formaldehyde in aliquots of one million cells each for 10 min at room temperature and combined after fixation. We performed the Hi-C assay following a standard protocol to investigate chromatin interactions within colorectal cancer cells^74^. HiC libraries were sequenced on an Illumina HiSeq 4000 with paired-end 75-bp read lengths. Paired-end HiC reads were aligned to hg19 genome with the HiC-Pro pipeline^75^. The pipeline was run with default settings, configured to assign reads to DpnII restriction fragments and to filter for valid pairs. The data were then binned to generate raw contact maps that then underwent iterative correction and eigenvector decomposition normalization to remove biases. The HiCCUPS function in Juicer^76^ was then used to call high-confidence loops. Visualization was done using Juicebox (https://aidenlab.org/juicebox/).

### Analysis of EIEs for repetitive element overlap

To assess the overlap of classes of repetitive elements with our identified EIEs, we obtained the ‘RepeatMasker’ and ‘Interrupted Repeats’ tracks from UCSC Genome Browser for hg19. For each EIE, we computed the fraction of the sequence that overlapped with the merged BED file containing the RepeatMasker and Interreputed Repeats annotations. We report the overlap separately for LINE, SINE and LTR repetitive element classes. Importantly, each EIE is exactly 1 kb long, so no length normalization is performed. To compute an expected proportion, we computed the fraction of hg19 covered by each repetitive element class. The results are reported in Fig. 1d and Extended Data Fig. 1a.

### Whole-genome sequencing with Oxford Nanopore

High-molecular-weight (HMW) genomic DNA was extracted from approximately 6 million COLO320DM cells using the Monarch HMW DNA Extraction Kit for Tissue (NEB #T3060L) following the Oxford Nanopore Ultra-Long DNA Sequencing Kit V14 protocol. After extracting HMW genomic DNA, we constructed Nanopore libraries using the Oxford Nanopore Ultra-Long DNA Sequencing Kit V14 (SQK-ULK114) kit according to the manufacturer’s instructions. We sequenced libraries on an Oxford Nanopore PromethION using a 10.4.1. Flow Cell (FLO-PRO114M) according to the manufacturer’s instructions. Basecalls from raw POD5 files were computed using Dorado (v.0.2.4).

### Identifying and remapping EIE-containing reads and detecting structural variants

We first identified Nanopore reads containing a single element by aligning reads with minimap2^77^ and filtered out reads that were not mapped by the algorithm (denoted by an asterisk in the RNAME column of the BAM entry). Then, taking these reads, we performed genomic alignment once again using minimap2 against hg19.

From these new alignments of only the reads found to contain the element under consideration, we performed two analyses for each element. First, we detected structural variant detection using Sniffles2^78^. Second, we identified overlap of reads with ecDNA-containing intervals that were reconstructed with long reads (see ‘Reconstruction of ecDNA amplicons with long-read data” section). In this second analysis (presented in Fig. 1f), we counted the number of reads covering regions contained with cycles reconstructed with CoRAL algorithm^31^. While this analysis does not explicitly distinguish reads originating from chromosomal versus extrachromosomal regions, we reasoned that elements carried on ecDNA would be amplified and therefore exhibit higher coverage; conversely, regions primarily chromosomal would show read counts similar to the overall genome coverage.

### Reconstruction of ecDNA amplicons with long-read data

We reconstructed ecDNA amplicons from ultralong Oxford Nanopore reads using the CoRAL algorithm^31^. In brief, this algorithm determines focally amplified regions of the genome using CNVkit^79^ and then finds reads that support this focally amplified region. In doing so, CoRAL identifies genomic breakpoints between the focally amplified seed region and disparate parts of the genome to create a ‘breakpoint graph’. From this breakpoint graph, putative ecDNA cycles are identified. We report the breakpoint graph in Fig. 1g, which includes a breakpoint between EIE 14 (annotated on chr3) and an intergenic region between *CASC8* and *MYC* on chr8.

In addition to detecting EIE 14 on the *MYC*-amplifying ecDNA in COLO320DM, we additionally quantified the number of reads that span a given EIE and any part of the COLO320DM genome amplified as ecDNA. We report the number reads that support an EIE as amplified on ecDNA in Fig. 1f.

In Extended Data Fig. 2b, we visualized reads connecting EIE 14 on chr3 with the chr8 ecDNA-amplified region using Ribbon (v 2.0.0)^80^.

### ATAC-seq analysis and normalization

ATAC-seq and ChIP-seq data for COLO320DM and SNU16 was obtained from ref. ^20^ and for PC3 and GBM39KT from ref. ^18^. Previously, ATAC-seq data were mapped to hg19. While ChIP-seq data were normalized to input, as input is not sequenced with ATAC-seq, these data were further normalized by library size. Specifically, ATAC-seq data were converted to a bedGraph format reporting the number of reads supporting each base position; these read densities were then normalized to parts per 10 million by dividing each position’s count by a normalization factor based on the total library size. These library-size-normalized data were used for downstream plotting

### TE old-versus-young classification

To classify TEs as old or young, we conducted a classification of EIE sequences listed in Supplementary Table 2. Elements were categorized based on their known evolutionary activity in humans. Young elements were defined as those from recently active subfamilies, including L1HS, L1PA2, SVA and AluY, which are known to have current or recent retrotransposition activity in the human genome. Classifications can be found in Supplementary Table 11.

### CRISPRi

The pHR-SFFV-dCas9-BFP-KRAB (Addgene, cat. no. 46911) plasmid was modified to dCas9-BFP-KRAB-2A-Blast as previously described^81^. Lentiviral particles were produced by co-transfecting HEK293T cells with the plasmid along with packaging plasmids psPAX2 and pMD2.G using a standard transfection method. Viral supernatants were collected at 48 and 72 h post-transfection, filtered through a 0.45-μm filter and concentrated by ultracentrifugation at 25,000 rpm for 2 h at 4 °C. Cells were transduced with lentivirus, incubated for 2 days selected with 1 μg ml^−1^ blasticidin for 10–14 days, and BFP expression was analysed by flow cytometry.

We took sgRNA specificity into account from the design phase of the CRISPRi screen. Our guide selection criteria included off-target scoring from ref. ^85^ and filtering. We designed the library in benchling (https://benchling.com) with multiple independent sgRNAs per EIE element. This redundancy helps to distinguish on-target biological effects from off-target noise. To increase our stringency and ensure that the effects of low-efficiency or low-specificity guides do not interfere with the interpretation of the screen, we used FlashFry^82^ to score our gRNAs with multiple tools (Supplementary Table 12) and specifically selected the CRISPRi specificity score developed by ref. ^83^ for filtering. We report effects only for elements with at least two guides achieving a specificity score greater than 0.2, which is the standard cut-off for this scoring parameter (similar to the Doench et al.^84^ cumulative distribution function score). The oligo pool encoding guides (Supplementary Table 10) were synthesized by Twist Bio and inserted into addgene Plasmid #52963 lentiGuide-Puro digested with Esp3I enzyme (NEB). The oligo pool was sequence validated. To investigate the effects of CRISPRi, we utilized a lentiviral delivery system to introduce sgRNAs into cells stably expressing the dCas9-KRAB repressor. Lentiviral particles were produced as described above. The viral titre was determined by transducing HEK293T cells with serial dilutions of virus and assessing transduction efficiency via flow cytometry for GFP expression.

For transduction, cells were seeded at a density of 1 × 10^6^ cells per well in six-well plates and transduced overnight with lentivirus at a low multiplicity of infection of 0.3, ensuring single sgRNA integration per cell. The following day, the medium was replaced with fresh growth medium. Two days post-transduction, cells were selected with 0.5 μg ml^−1^ puromycin for 4 days to enrich successfully transduced cells. GFP expression was monitored by flow cytometry to assess transduction efficiency. After selection, cells were collected at multiple timepoints: baseline (day 4 after transduction), day 3, week 1 and month 1 (30 days). Genomic DNA was extracted using the DNeasy Blood & Tissue Kit (Qiagen) following the manufacturer’s instructions.

Integrated sgRNA sequences were amplified from genomic DNA using a multistep PCR process. First, sgRNA cassettes were amplified using Primer set 1: hU6_pcr_out_fw (tggactatcatatgcttaccgtaacttgaaagt) and efs_pcr_rev (ctaggcaccggatcaattgccga). PCR reactions contained 0.8 μl each of 25 μM primers, 1–2 μg genomic DNA, water and 25 μL NEB 2x master mix in a total volume of 50 μl. PCR conditions included an initial 3 min at 98 °C, followed by 15–17 cycles of 20 s at 98 °C, 20 s at 58 °C and 30 s at 72 °C, concluding with a final extension for 1 min at 72 °C. PCR products (~400 bp) were verified by gel electrophoresis and purified. The second PCR step added Illumina sequencing adapters using primers (P5 stagger -hu6 and p7adpt_spRNAl105nt_rev). Reactions contained 10–50 ng of purified PCR1 product, 0.8 μl of each primer, water and 25 μl of NEB 2× master mix in a total volume of 50 μl. PCR conditions were: initial denaturation for 30 s at 98 °C, followed by six cycles of 15 s at 98 °C, 15 s at 60 °C and 30 s at 72 °C, with a final extension of 1 min at 72 °C. PCR products (200–300 bp) were gel-verified and purified using AMPure XP beads. A final indexing PCR step was performed using Truseq-based P5 and P7 indexing primers. Reactions contained 10–50 ng DNA from PCR2, 0.8 μl of each primer, water and 25 μl NEB 2× master mix in 50 μl total volume. Conditions included 30 s at 98 °C followed by six cycles of 15 s at 98 °C, 15 s at 63 °C and 30 s at 72 °C, ending with a 1-min extension at 72 °C. Products were purified with AMPure XP beads and sequenced on an Illumina NextSeq platform using single-end 50-bp reads. Sequencing data were processed to quantify sgRNA representation at each timepoint, allowing analysis of sgRNA abundance dynamics over the experiment duration.

### CRISPRi fitness screen analysis

To compute the effect of each guide on cell fitness, we first quantified guide counts from sequencing libraries. To normalize counts across libraries, we converted raw guide counts to counts per million (CPM) and retained guides that had CPM values of at least 20 across all days tested. We also filtered out guides with high off-target scores (Supplementary Table 12, 0.2 cut-off from optimized CRISPRi design parameters^83^) and excluded EIEs with fewer than two guides remaining after filtering. After confirming that normalized guide abundances were robust across replicates, we proceeded with our analysis using the average of guide replicates at each timepoint. We next scored the relative fitness of each guide against the NTC by computing the ratio of CPM values between a guide and the NTC at the particular timepoint. Finally, we transformed this distribution to *Z* scores and reported this as the relative fitness effect of each guide.

### CRISPR-CATCH

In our study, we used the CRISPR-CATCH technique to isolate and analyse ecDNA structures. Following the standard protocol^22^, we designed two sgRNAs targeting specific enhancer regions: sgRNA #1 (ATATAGGACAGTATCAAGTA) and sgRNA #2 (TATATTATTAGTCTGCTGAA). These sgRNAs directed the Cas9 nuclease to introduce double-strand breaks at the targeted sites, linearizing the circular ecDNA molecules. The linearized DNA was then subjected to PFGE using *Saccharomyces cerevisiae* and *Hansenula* *wingei* DNA ladders as molecular weight markers to facilitate size-based separation. Distinct DNA bands corresponding to the targeted ecDNA were excised from the gel for downstream analyses, including sequencing.

### Probe design

Probes were designed against human genome assembly hg19, tiling the regions in Supplementary Table 7 using the probe designing software described previously^36,37^. We restricted the selection of the 40-mer probe targeting regions to a GC content between 20% and 80% and a melting temperature of 65–90 °C, and excluded sequences with non-unique homology—defined as sharing a 17-mer or longer sequence with other genomic regions—or homology to common repetitive elements in the human genome listed in RepBase, using a 14-mer cut-off. Targeting probes were then appended with a 20-mer barcode per target region. Probe design software is available via GitHub at https://github.com/BoettigerLab/ORCA-public. Finalized probe libraries were ordered as an oligo-pool from GenScript.

### ORCA imaging

ORCA hybridization was performed as previously described^36,37^. In brief, 40-mm Bioptechs coverslips were prepared with EMD Millipore poly-D-lysine solution (1 mg ml^−1^, 20 ml, dilute 1:10) (Sigma, cat. no. A003E) for 40 min. Coverslips were then rinsed three times in 1× PBS. Cells were passaged onto the coverslips and allowed to adhere overnight. The next day, the coverslip with cells were rinsed three times in 1× PBS and then fixed for 10 min in 4% paraformaldehyde. For DNA imaging, cells were then permeabilized in 0.5% Triton-X 1× PBS for 10 min followed by 5 min of denaturing in 0.1 M HCl. A 35-min incubation in hybridization buffer prepared samples for the primary probe. Primary probes were added (1 μg) directly to the sample in hybridization solution, and then the sample was heated to 90 °C for 3 min. An overnight 42 °C incubation (or at least 8 h incubation) was performed, followed by post-fixation in 8% paraformaldehyde + 2% glutaraldehyde in 1× PBS, before being stored in 2× SSC or used immediately for imaging. For RNA imaging, the HCl, heat and post-fixation steps were omitted.

DNA samples were imaged on one of two different homebuilt set-ups designed for ORCA, ‘scope-1’ or ‘scope-3’, depending on instrument availability. Microscope design parameters were deposited in the Micro-Meta App^85^. The design and assembly of the scope-1 system is described in detail in our prior protocol paper^37^. Both systems use a similar auto-focus system, fluidics system and scientific complementary metal-oxide-semiconductor camera (Hamamatsu FLASH 4.0), although scope-3 had a larger field of view (2,048 × 2,048 108-nm pixels) compared with scope-1 (1,024 × 1,024 154-nm pixels).

RNA samples were imaged on a different homebuilt set-up designed for ORCA designated as the ‘Yale lumencor system’. This system uses a similar auto-focus system and fluidics system, with a scientific complementary metal-oxide-semiconductor camera (Hamamatsu ORCA BT fusion) with a field of view of 2,304 × 2,304 at 108 nm per pixels and an Olympus PlanApo 60× objective.

Automated fluidics handling is described in detail in our prior protocol paper^37^. In brief, fluid exchange between each imaging step was performed by a homebuilt robotic set-up. The system used a three-axis computer numerical control router engraver, buffer reservoirs and hybridization wells (96-well plate) on a three-axis stage, ethylene tetrafluoroethylene tubing, imaging chamber (FCS2, Bioptechs), a needle and peristaltic pump (Gilson F155006). The needle was moved between buffers or hybridization wells and transported across the samples through tubing using a peristaltic pump. Open-source software for the control of the fluidics system is described in the ‘Software availability’ section.

Sequential imaging of ORCA probes was conducted alternating between hybridization of fluorescent adapter probes, readout probes complementary to the barcodes on the primary probe sequences, imaging and stripping of probes, as described previously^36,37^. In brief, a *z*-stack spanning 10 μm was acquired with 250 nm step sizes, alternating laser excitation between the data channel and fiducial marker at each step. Readout probes were labelled with Alexa-750 fluorophores. The fiducial probe was labelled in cy3 and added only in the initial round. RNA imaging was performed with the EIE 14 probe labelled with the Alexa-750 and the *MYC* probe labelled with the Cy5 fluorophores.

Sequence for the fiducial: /5Cy3/AGCTGATCGTGGCGTTGATGCCGGGTCGAT

Sequence of Cy5: /5Cy5/TGGGACGGTTCCAATCGGATC

Sequence of the 750:/5Alex750N/ACCTCCGTTAGACCCGTCAG

### Image processing

Image processing was performed with custom MATLAB functions available via GitHub at https://github.com/BoettigerLab/ORCA-public. In brief, cells were maximum projected, and pixel-scale alignment across all fields of view was computed using the fiducial signal. This alignment was then applied in three dimensions across all 250-nm *z* steps. Cellpose^86^ was then used to segment individual cells. A cell-by-cell fine scale (subpixel) alignment was then computed, and aligned individual cells were then ready for 3D-spot calling. The individual ecDNA spots and their 3D positions computed to subpixel accuracy using the corresponding raw 3D image stacks and the 3D DaoSTORM function in storm-analysis toolbox (10.5281/zenodo.3528330), an open-source software for single-molecule localization, adapted for dense and overlapping emitters following the DaoSTORM algorithm^87^. DaoSTORM was run in the 2D-fixed mode, as the 3D fitting modes are for estimating axial position from astigmatism in the *xy* plane, rather than computing it directly from a *z*-stack. The fixed-width point spread function of the microscope is precomputed using 100-nm (subdiffraction) fluorescent beads. A minimum detection threshold of 30 sigma was used for the fit. The *z*-position of the localizations was computed using Gaussian fit to the vertically stacked localizations, with an axial Gaussian width also precomputed from *z*-stack images with 100-nm fluorescent beads. Additional information can be found in the read-the-docs for storm-analysis at https://storm-analysis.readthedocs.io/en/latest/.

### Minimum pairwise distance quantification

All pairwise distances between genomic regions were calculated on a per-cell basis. The shortest distances were saved for each *MYC* centroid and EIE 14 and *PVT1* such that each *MYC* centroid has one corresponding shortest distance per EIE 14 and *PVT1*. For each cell, a sphere radius *r* *=* 4um (the average radius of cells calculated with Cellpose mask) with randomly simulated points corresponding to the number of *MYC*, EIE 14 and *PVT1* centroids. The same minimum pairwise distance quantification was calculated on the randomly simulated points.

### Ripley’s *K* quantification

To calculate the density-corrected distance ratios, a distance cut-off of 2 μm and an interval density of 0.01:0.01:2 was used. The spatial relationship between *MYC* and EIE 14 and *MYC* and *PVT1* were quantified as follows. On a per-cell basis, the distance density function was calculated, truncated at the specified cut-off. A uniform distribution was then computed over the same interval, and a ratio of these values was taken. This ratio was then corrected by the volume of the interval shell.

### Reporter plasmid construction and transfection

All plasmids are made with Gibson assembly (NEB HIFI DNA assembly kit) according to the manufacturer’s protocol. We used a plasmid from this publication^20^ containing the *MYC* promoter (chr8:128,745,990–128,748,526, hg19) driving NanoLuc luciferase (PVT1p-nLuc) and a constitutive thymidine kinase (TK) promoter driving Firefly luciferase. This plasmid served as the negative control. pGL4-tk-luc2 (Promega) plasmids with an enhancer (chr8:128347148–128348310) were used as the positive control^20^. In the test plasmid, the *cis*-enhancer was replaced by 1.7 kb sequence of EIE 14 or by Part #1: L1PA2 or by Part #2: L1M4a1 (Supplementary Table 13). To assess luciferase reporter expression, COLO320DM cells were seeded into a 24-well plate with 100,000 cells per well. Reporter plasmids were transfected into cells the next day with Lipofectamine 3000 following the manufacturer’s protocol, using 0.25 μg DNA per well. Luciferase levels were quantified using Nano-Glo Dual reporter luciferase assay (Promega).

### Statistics and reproducibility

All statistical tests used, replicate information and sample size information are reported in the figure legends. No statistical method was used to predetermine sample size. No samples or data points were excluded. The experiments were not randomized. The investigators were not blinded to the conditions of the experiments during data analysis.

### Reporting summary

Further information on research design is available in the Nature Portfolio Reporting Summary linked to this article.

## Online content

Any methods, additional references, Nature Portfolio reporting summaries, source data, extended data, supplementary information, acknowledgements, peer review information; details of author contributions and competing interests; and statements of data and code availability are available at 10.1038/s41556-025-01788-6.

## Supplementary information

Reporting Summary

Supplementary Tables Supplementary Table 1. EIE sequence from HiC hg19. This table includes genomic sequences identified from Hi-C analyses aligned to the hg19 genome reference. Supplementary Table 2. EIE from T7 in RepBase, describing genomic regions matching known elements from RepBase database. Columns: ‘Class’: classification of the element (for example, NonLTR/SINE). ‘Dir’: direction of element integration. ‘Sim’: similarity percentage to reference sequences. ‘Score’: alignment score indicating match quality. Supplementary Table 3. RepBase Ref for T4. Reference annotations from RepBase database used in T4. Supplementary Table 4. Nanopore seq SV EIE14 hg 19. Structural variant (SV) details identified by Nanopore sequencing aligned to the hg19 genome. Columns: ‘REF’: reference allele. ‘ALT’: alternative allele indicating the variant or sequence. ‘SVLEN’: length of the structural variant. ‘INFO‘: variant details, for example, SVTYPE (type of variant). Supplementary Table 5. Nanopore seq SV EIE14 hg 38. Similar to T4 but aligned to the hg38 genome. Supplementary Table 6. EIE 14 sequence. Supplementary Table 7. ORCA probes. ORCA primary probes for RNA and DNA FISH imaging. Each primary probe has two sequences corresponding to forward and reverse primer index, a common readout sequence, a unique readout sequences and the 40-bp sequence with homology to target. Supplementary Table 8. CRISPRi zscore all gRNAhg19. CRISPRi results showing *Z* scores for individual guide RNAs. Columns: ‘Baseline_zscore’, ‘3days_zscore’, ‘2weeks_zscore’, ‘1month_zscore’: *Z* scores measured at different timepoints. Supplementary Table 9. CRISPRi zscore combinedhg19. Supplementary Table 10. CRISPRi guide RNA hg19. Details of guide RNAs used in CRISPRi experiments. Supplementary Table 11. Classified TE old new. Classification of TEs as old or young based on evolutionary activity. Columns: ‘TE_Age’: Indicates if TE is considered ‘old’ (inactive) or ‘young’ (recently active). Supplementary Table 12. SGRNA OFF TARGET. Analysis for off-target effects for sgRNAs used based on different publications indicated in columns. Supplementary Table 13. Enhancer seq luciferase. Sequences of enhancer elements tested in luciferase reporter assays, separated into two parts (L1PA2 and L1M4a1).

## Source data

Source Data All Figures Source Data Fig. 1d and Extended Data Fig. 1a: statistical source data related to Fig. 1d and Extended Data Fig. 1a. Source Data Fig. 1f: statistical source data related to Fig. 1f. Source Data Fig. 5c: statistical source data for Fig. 5c. Source Data Fig. 3 and Extended Data Fig. 3: statistical source data related to Fig. 3 and Extended Data Fig. 3. Source Data Fig. 1f: statistical source data related to Fig. 1f. Source Data Fig. 1d and Extended Data Fig. 1a: statistical source data related to Fig. 1d and Extended Data Fig. 1a. Source Data Extended Data Fig. 5c: statistical source data related to Extended Data Fig. 5c. Source Data Extended Data Fig. 6a,b,c. Statistical source data related to Extended Data Fig. 6.

Source Data Fig. 2 Unprocessed gel related to Fig. 2b.

Source Data Fig. 5 Unprocessed images related to Fig. 5b.

Source Data Extended Data Fig. 3/Table 3 Unprocessed image related to Extended Data Fig. 3a and statistical source data for Extended Data Fig. 3.

## Data Availability

All sequencing data generated in this study are available via Gene Expression Omnibus (GEO) accession number GSE277492 at https://www.ncbi.nlm.nih.gov/geo/query/acc.cgi?acc=GSE277492 and via BioProject NCBI ID: 1162466 at https://www.ncbi.nlm.nih.gov/bioproject/1162466. Raw RNA imaging data related to Fig. 5 are available via Zenodo at 10.5281/zenodo.16921322 (ref. ^88^). All raw imaging data related to the DNA are available upon request as they are large. The processed data tables from image analysis recording *x*,*y*,*z* positions of RNA and DNA are available via GitHub at https://github.com/sedona-Eve/Kraft_Murphy_Jones_ecDNA/. Source data are provided with this paper.

## Code availability

The image analysis code is publicly available via GitHub at https://github.com/BoettigerLab/ORCA-public/ and at https://storm-analysis.readthedocs.io/en/latest/analysis.html. Code for reconstructing amplicons from long-read data with the CoRAL algorithm is available via GitHub at https://github.com/AmpliconSuite/CoRAL.
