# Supplementary figures and images for: Enhancer activation from transposable elements in extrachromosomal DNA
Source: Nat Cell Biol. 2025 Oct 21;27(11):1914–24. doi: 10.1038/s41556-025-01788-6 (PMC12611757; doi:10.1038/s41556-025-01788-6)

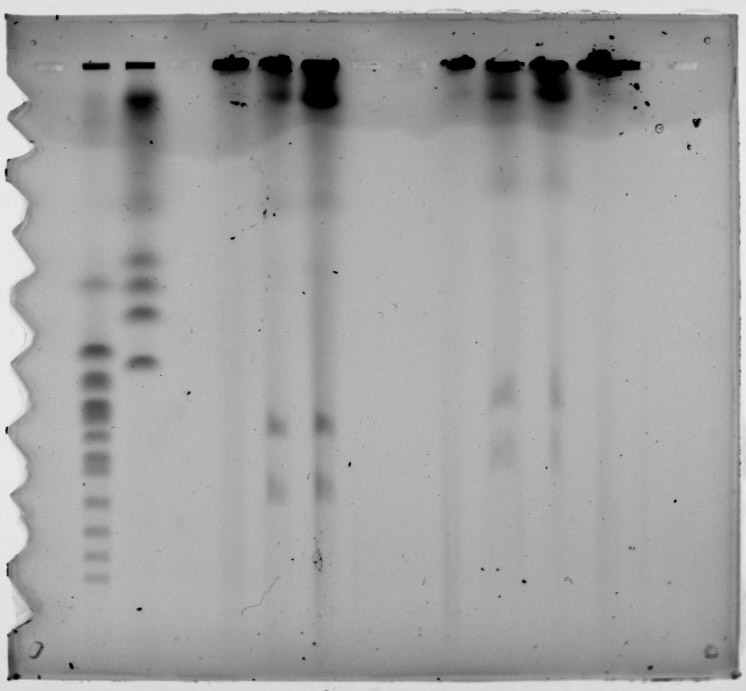

Supplement: Supplementary file 4 — Unprocessed gel related to Fig. 2b. [file 41556_2025_1788_MOESM4_ESM.jpg]

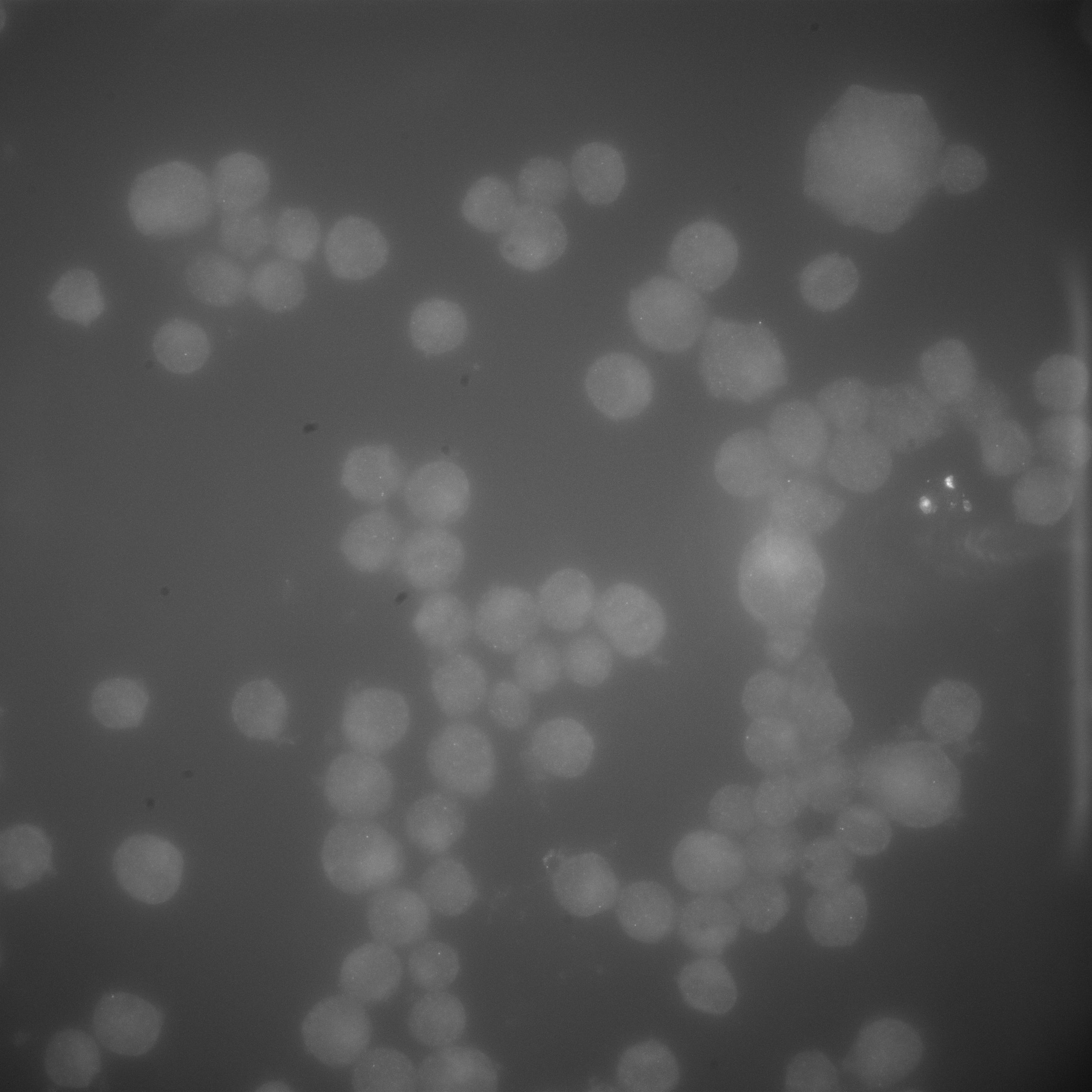

Supplement: Supplementary file 6 — Unprocessed image related to Extended Data Fig. 3a and statistical source data for Extended Data Fig. 3. [file 41556_2025_1788_MOESM6_ESM.jpg]
