# Supplementary material for: Enhancer activation from transposable elements in extrachromosomal DNA
Source: Nat Cell Biol. 2025 Oct 21;27(11):1914–24. doi: 10.1038/s41556-025-01788-6 (PMC12611757; doi:10.1038/s41556-025-01788-6)

Source Data Figure 5B E14 DM

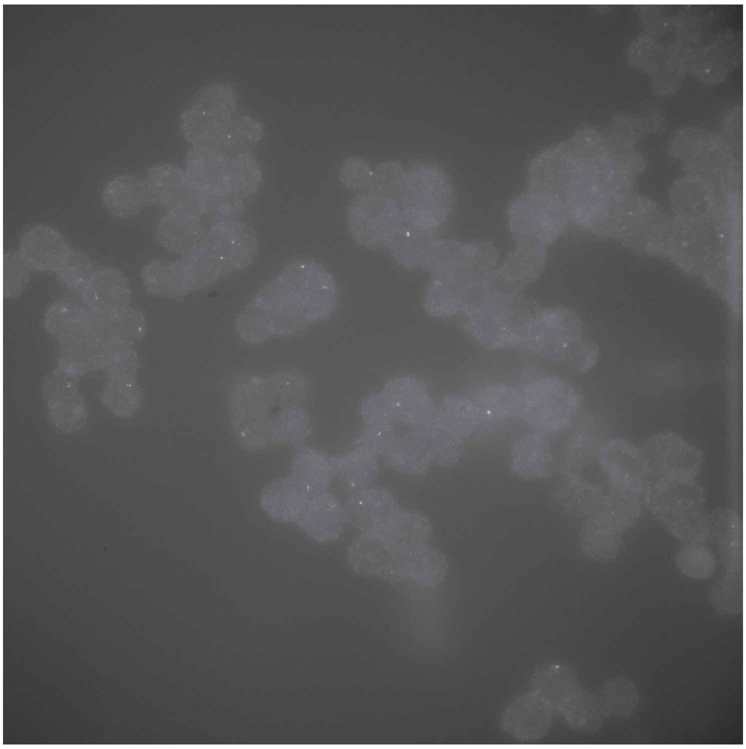

Source Data Figure 5B E14 HSR

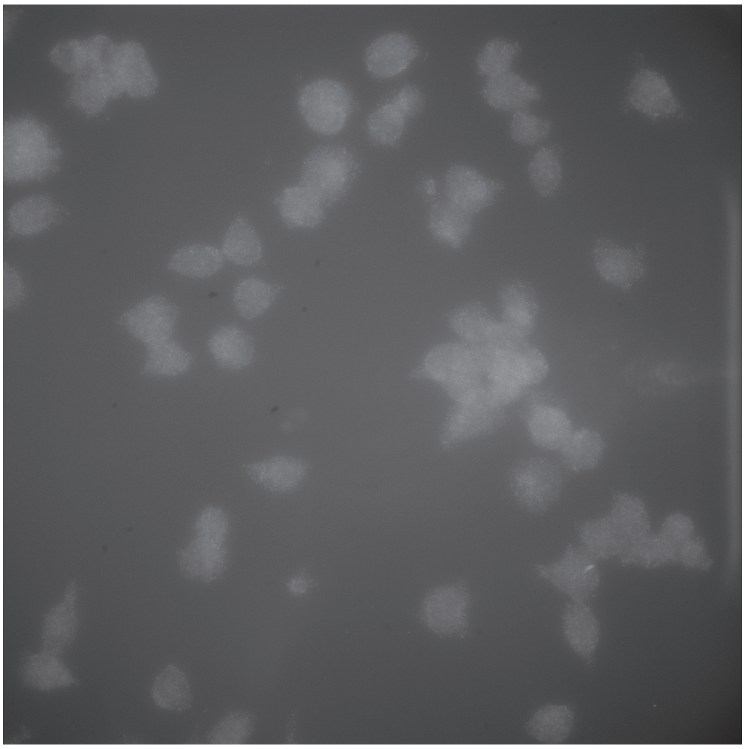

Source Data Figure 5B *MYC* DM

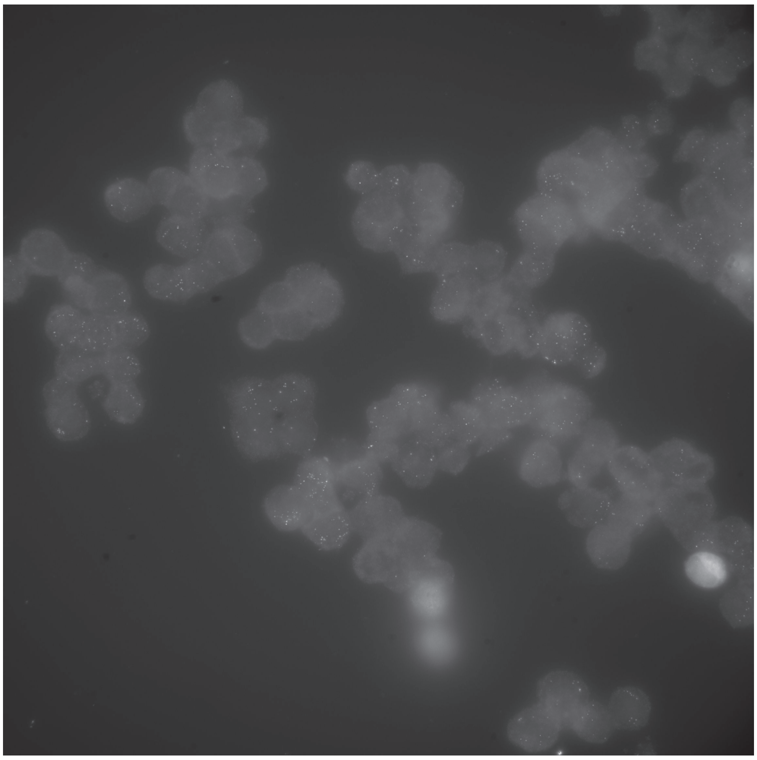

Source Data Figure 5B *MYC* HSR

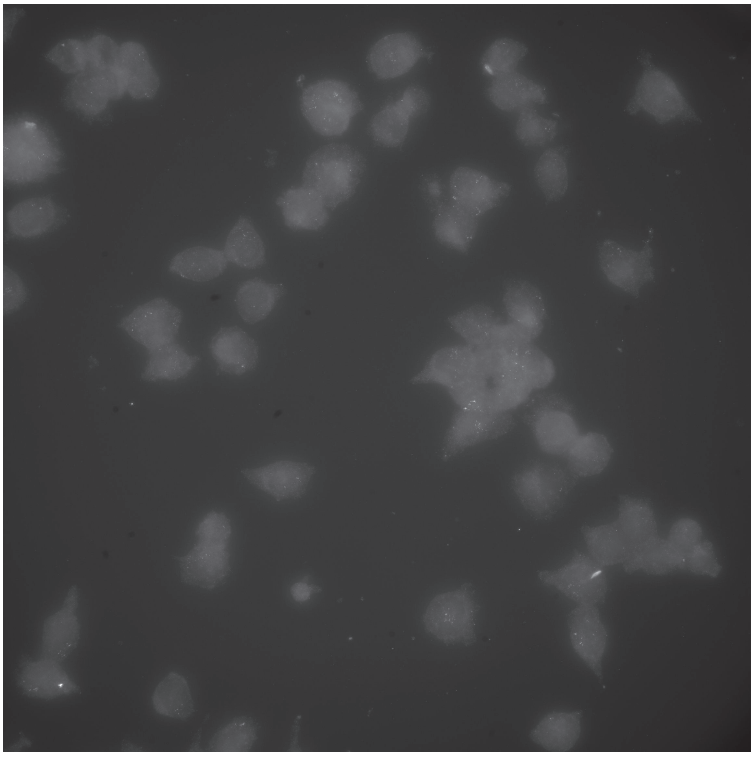

Supplement: Supplementary file 5 — Unprocessed images related to Fig. 5b. [file 41556_2025_1788_MOESM5_ESM.pdf]
